# Surgical Outcome of Medial Rectus Resection in Recurrent Exotropia: A Novel Surgical Formula

**DOI:** 10.1155/2015/758463

**Published:** 2015-03-17

**Authors:** Abbie Sheung-Wan Luk, Jason Cheuk-Sing Yam, Henry Hing-Wai Lau, Wilson Wai-Kuen Yip, Alvin Lerrmann Young

**Affiliations:** Department of Ophthalmology & Visual Sciences, 1/F Eye Centre, The Chinese University of Hong Kong, Prince of Wales Hospital, Shatin, Hong Kong

## Abstract

*Purpose*. To evaluate the surgical outcomes of unilateral or bilateral medial rectus (MR) muscle resection for recurrent exotropia after bilateral lateral rectus (BLR) muscle recession based on a novel surgical formula. *Methods*. Forty-one consecutive patients with unilateral or bilateral MR muscle resection for recurrent exotropia after BLR muscle recession were included in this retrospective study. All surgeries were performed according to the formula: 1.0 mm MR muscle resection for every 5 prism dioptres (PD) of exotropia, with an addition of 0.5 mm to each MR muscle operated on. *Results*. The mean recurrent exotropia distant deviation was 28 PD ± 11.2 (range 14 to 55 PD). Overall at postoperative 1 month, 36 (88%) achieved successful outcomes, 4 (10%) had undercorrection, and 1 (2%) had overcorrection. At postoperative 6 months, 29 (71%) achieved successful outcomes, 12 (29%) had undercorrection, and none had overcorrection. Subgroup analysis showed no statistically significant difference in success rates between unilateral and bilateral MR groups. *Conclusion*. Unilateral or bilateral MR muscle resection using our surgical formula is a safe and effective method for calculating the amount of MR resection in moderate to large angle recurrent exotropia, with a low overcorrection rate.

## 1. Introduction

Reoperation in strabismus surgery is not uncommon for patients with under- or overcorrection of an ocular deviation [1, 2]. In cases of recurrent exotropia after primary lateral rectus (LR) muscle recession surgery; there remains a choice of conservative treatment or reoperation, depending on the severity of deviation. Debate still exists as to whether LR muscle rerecession or medial rectus (MR) muscle resection would be the most optimal treatment. Some advocate that LR muscle rerecession can help conserve the unoperated MR muscles and has comparable surgical outcomes with MR resection [3]. The advantage of graded resection surgery is that the surgeon can work with muscles not previously operated on, which is a technically easier procedure with clearer surgical landmarks. However, deduction of optimal MR muscle resection for recurrent exotropia after two-muscle surgery remains controversial, as current surgical dosage tables [4] for muscle resection calculations are suggested for primary operation, with unpredictability in outcomes for reoperations. Variable results are reported in bilateral MR muscle resection for recurrent exotropia despite similar surgical dosages adopted using these surgical tables, with success rates ranging from 54% to 83% [3, 5]. As recurrent exotropia is commonly seen after primary operation for intermittent exotropia [6], a surgical formula for reproducing predictable results in reoperations would be beneficial. Lau et al. [7] reported their approach of unilateral 1.0 mm MR muscle resection for every 5 prism dioptres (PD) exotropia in single-staged three-muscle surgery for large angle exotropias, with a 25% undercorrection rate. In this study we adopted a surgical formula of 1.0 mm for every 5 PD, with addition of 0.5 mm on each operated medial rectus muscle for MR resection in recurrent exotropia after bilateral LR recession, and report our surgical results.

## 2. Materials and Methods

Our retrospective study included 41 consecutive patients who underwent unilateral or bilateral MR muscle resection for recurrent exotropia. Ethics committee approval was granted. Exclusion criteria included patients with paralytic, restrictive, or other ocular or neurological disorders, infantile exotropia, poor vision, or amblyopia. All patients had a history of bilateral lateral rectus (BLR) muscle recession as the primary surgery for basic type intermittent exotropia performed at our hospital between January 2007 and December 2012. Patients underwent complete ophthalmological examination prior to both the first BLR muscle recession and second MR muscle resection surgery. The age at each operation, gender, visual acuity, preoperative near and distant deviation, postoperative complications, and postoperative near and distant angle measurement at postoperative week 1, month 1, and month 6 and at last follow-up were recorded from medical records. Recurrence was defined as an alignment of more than 10 PD exotropia in those who had more than 3-month interval after initial BLR muscle surgery. Conservative management of convergence exercises was encouraged during the interval period between surgeries. Surgery for subsequent MR muscle resection was undertaken when the parents or guardian were keen for strabismus reoperation with the angle of deviation of at least 15 PD exotropia. Preoperative and postoperative angle deviations were measured by prism alternate cover tests at near (33 cm) and distant (6 m) targets. Stereoacuity pre- and postoperatively was measured for patients at near by using TNO random dot stereo test (Lameris Instrumenten, Utrecht, Netherlands) with red-green glasses.

We adopted a surgical formula for every 5 PD exotropia to 1.0 mm of medial rectus muscle resection, with addition of 0.5 mm to each operated medial rectus muscle. All surgeries were performed under general anaesthesia by two paediatric ophthalmologists. For the first operation of LR muscle recession, Wrights' table [4] was used to measure the muscle recession from muscle insertion. For the second operation of both unilateral and bilateral MR muscle resection, our surgical formula was adopted. Both muscle recession and resection surgeries were done using scleral fixation sutures with 6-O vicryl, with conjunctival wounds closed with 8-O vicryl. For MR muscle resection, we used two double-armed 6-O vicryl sutures to secure the muscle to the sclera to minimize sagging. No adjustable sutures were used.

Successful surgical outcome was defined as orthophoria, esodeviation equal to or less than 5 PD, or exodeviation equal to or less than 10 PD at distance. The surgical outcomes at postoperative 1 week, 1 month, and 6 months and at last follow-up were reported. Statistical analysis was done using SPSS (version 19.0, SPSS Inc, Chicago, Illinois, USA). The chi-square test, Mann-Whitney *U* test, and Fisher's exact test were used for determining relationships between groups for preoperative characteristics and surgical outcomes. All tests were considered to be statistically significant if *P* value was <0.05.

## 3. Results

There were 41 patients, with 16 males and 25 females. The mean age of first onset of intermittent exotropia was 4.7 ± 4.4 years old (range 0.5–25 years). The mean age at bilateral LR muscle recession surgery was 9.3 ± 8.9 years old (range 1.3–42 years), and the mean LR muscle recessed at each eye was 7.5 mm (range 4.5–9.5 mm). Thirty-four (83%) patients had intermittent exotropia and 7 (17%) had constant exotropia due to decompensated intermittent exotropia. There were 19 (46%) unilateral MR muscle resections and 22 (53%) bilateral MR muscle resections. The mean age at MR muscle resection surgery was 12.2 ± 8.6 years old (range 2.1–43 years). Indications for unilateral MR muscle resection included patients with preoperative distant exotropia less than or equal to 25 PD, and those above 25 PD underwent bilateral MR resection. The overall mean preoperative deviation prior to reoperation was 28 PD ± 11.2 (range 14–55 PD) for distance and 30 PD ± 11.3 (range 10–55 PD) for near. Of the 41 patients, 8 (19.5%) underwent additional procedure for oblique muscles during the same operation, 4 with unilateral and 4 with bilateral inferior oblique recession. The overall mean follow-up time for all patients was 17.5 ± 12 months (6–46 months). Within the unilateral MR muscle resection group, 8 patients (42%) had prior bilateral LR muscle recession of over 7 mm in each eye, compared with 16 (73%) in the bilateral MR muscle resection group. The preoperative patient data comparing unilateral and bilateral medial rectus muscle resection are summarized in Table 1.

### 3.1. Surgical Outcomes

For immediate surgical outcomes, the overall success rate at postoperative 1 week was 90% (37 of 41 patients), with 1 (2%) overcorrection and 3 (7%) undercorrections. For short-term outcomes, the overall success rate was 88% (36 of 41 patients) at postoperative 1 month, with 1 (2%) overcorrection and 4 (10%) undercorrections. At last follow-up, there were 28 (68%) successful outcomes, 13 (32%) undercorrections and no overcorrections. Comparison of success rates between unilateral and bilateral groups is shown in Table 2. Subgroup analysis showed no statistically significant difference in success rates between unilateral and bilateral MR muscle resection groups at postoperative 1 month (*P* = 0.115) and 6 months (*P* = 0.763) and at last follow-up (*P* = 0.511). There were no postoperative complications noted throughout this period.

Of the 4 patients with undercorrection at postoperative 1 month, 1 had unilateral and 3 had bilateral MR muscle resection. One patient had concomitant bilateral MR muscle resection with bilateral inferior oblique recessions in the same operation. Further analysis shows that these patients had a large mean distance deviation angle of 52.5 ± 15 PD (range 45–75 PD) at primary surgery. At the second surgery, the mean recurrent exotropia distance deviation was 36.3 ± 14.4 PD (range 25–55 PD), which is higher than the overall mean deviation of 28 PD.

### 3.2. Stereopsis

Stereopsis using TNO random dot test at near was compared between pre- and post-MR muscle resection surgery. At last follow-up, good stereoacuity of 60 seconds of arc or better was found in 12 of 41 patients (29%). Overall, improvement in stereopsis compared with baseline was found in 11 patients (27%), no change was found in 29 (71%), and decrease in stereopsis was found in 1 (2%).

## 4. Discussion

Exotropia has been shown to be more prevalent than esotropia in our Hong Kong Chinese population, compared with other studies with predominantly white populations [8]. Recurrent exotropia after first surgery for intermittent exotropia is therefore not uncommon in our locality. For surgical outcomes of intermittent exotropia, highly variable success rates have been reported in literature ranging from 42 to 81% [9]. In Asian patients, Yam et al. [6] reported success rate of 51% for intermittent exotropia after bilateral LR recession at 3 years in a Chinese population, whereby Choi et al. [2] reported a success rate of 58% at a comparable 3.8-year follow-up in a Korean population. Furthermore, variable success rates have also been reported in the limited studies on reoperation for recurrent exotropia ranging from 66 to 83% [3, 5], with recurrence rates after reoperation reported to be 33% by 18 months and 60% by 51 months [10]. However, the success rate of these studies may not be directly comparable due to differences in preoperative angle deviation and surgical technique.

There is currently no normogram in literature to determine the surgical dosage for patients with recurrent exotropia. Several studies report variable MR muscle resection correction ranging from of 2 to 6.7 PD per mm [3, 11–13], but no easily reproducible or predictive surgical formula has yet been proposed. Herein, we report an easy approach using our surgical formula of MR muscle resection for recurrent exotropia, which can help simplify the calculation of the amount of MR muscle to be resected. As in the primary surgery for intermittent exotropia [6], significant postoperative exodrift was also noted in our results after postoperative month 1. The success rate of repeat surgery may tend to decrease with time and studies on further long-term outcome are needed. These findings are in agreement with the accepted view that there is a tendency towards exodrift with increasing time postoperatively [1, 14, 15]. Furthermore, the cause and pathophysiology of exodrift remain unknown.

For patients with smaller angles of exotropia (i.e., distance deviation less than or equal to 20 PD), unilateral MR muscle resection can be adopted. Advantages of one muscle surgery include less surgical time required, lower risk of surgical complications, and potential future use of fellow muscle for reoperations in cases of recurrence [12]. Chae et al. [13] reported good surgical outcomes of 80% success at 6 months postoperatively with unilateral MR muscle resection after BLR muscle recession, with use of on average 3.53 PD per millimeter of MR muscle resection correction. However, their study included only patients with intermittent exotropia and the intraoperative adjustment of muscle resection was variable between patients. Large unilateral MR muscle resection was reported to be superior to bilateral MR muscle resection in a study by Yang and Hwang [5], whereby the success rate at the last follow-up of 22 months postoperatively was 80% in the unilateral group compared with 54% in the bilateral group with a combined success rate of 66%, of which our results are comparable with similar last follow-up time. Their rate of overcorrection was 10% for the unilateral group and 42% for the bilateral group, which was significantly higher than our study results of no overcorrections in both groups at last follow-up. Furthermore, none of our patients required base-out prisms for overcorrection. It is of interest that in their study the amount of unilateral MR muscle resection was unusually high, from 7.0 mm to 10.0 mm, even for recurrent exotropia up to 25 prism diopters. Conversely, the average amount of MR muscle resection in our study was 4.6 mm ± 0.8 (range 3 to 6 mm). This may be due to different surgical technique in performing the resection. However, in our current method we can preserve more muscle and also minimize the potential risk of introducing restriction and lateral incomitance.

An alternative to MR muscle resection would be LR rerecession surgery. Yazdian and Ghiassi [16] reported high success rates from bilateral LR muscle rerecession of up to 17 mm, from the limbus for recurrent exotropia without producing significant limitation in abduction. However, this was only a small study size and there remain limited studies in literature on LR muscle rerecession outcomes. Choice of surgical treatment often depends on the primary surgery undertaken and the surgical experience in different centers.

For reoperations in recurrent exotropia, we believe that our study is meaningful in proposing a surgical formula that can be useful for both unilateral and bilateral MR muscle resection. Firstly, it can be adopted for relatively larger angle exotropias, as our overall mean distant preoperative deviation prior to MR muscle resection was 28 PD (with mean of 35.5 PD and up to 55 PD in the bilateral MR muscle resection group), which is higher than other reported case series [5, 13]. Secondly, our study shows a good overall immediate surgical success rate of 88% with no overcorrections noted by postoperative 6 months in all patients. This is important as to minimizing any loss in stereopsis induced from significant consecutive esotropia. Lastly, our surgical formula is simple to use and can be easily adopted in clinical practice, as shown in Table 3. Limitations of this study include its retrospective nature and small study size. Further prospective studies in the future for adjustment of the surgical formula may be useful for optimizing the surgical outcome and in formulating a predictable normogram.

## Figures and Tables

**Table 1:** Preoperative patient characteristics.

	UMR resection (*n* = 19)	BMR resection (*n* = 22)	*P* value
Mean age of onset (years)	3.4 ± 2.1	5.8 ± 5.5	0.129
Gender: male	7 (37%)	9 (41%)	0.790
Mean preoperative deviation at BLR recession			
Near (PD)	31.2 ± 11.7 (12 to 55)	45.0 ± 10.6 (25 to 65)	0.001
Distant (PD)	32.5 ± 10.6 (18 to 55)	44.1 ± 12.3 (25 to 75)	0.004
Mean age at BLR recession (years)	7.8 ± 8.6	10.5 ± 9.2	0.270
Mean age at MR resection (years)	11.5 ± 8.2	12.8 ± 9.0	0.590
Interval between operations (months)	48.3 ± 29.4 (10 to 144)	29.1 ± 23.1 (6 to 85)	0.010
Mean preoperative deviation for MR resection			
Near (PD)	21.9 ± 5.4 (10 to 30)	36.7 ± 10.5 (18 to 55)	0.000
Distant (PD)	19.4 ± 3.8 (14 to 25)	35.5 ± 10.1 (30 to 55)	0.000
Presence of oblique surgery	6 (31.6%)	2 (9%)	0.070
Follow-up duration (months)	20.4 ± 12.5 (6 to 45)	18.7 ± 13.3 (6 to 46)	0.517

UMR: unilateral medial rectus; BMR: bilateral medial rectus; PD: prism dioptres; BLR: bilateral lateral rectus; LR: lateral rectus.

**Table 2:** Success rates between unilateral and bilateral medial rectus muscle resection groups at postoperative 1 month and 6 months and at last follow-up.

	UMR resection (*n* = 19)	BMR resection (*n* = 22)	*P* value
Success (%)			
Postoperative 1 week	19 (100%)	18 (82%)	0.05
Postoperative 1 month	18 (95%)	18 (82%)	0.115
Postoperative 6 months	13 (68%)	16 (73%)	0.763
At last follow-up^a^	12 (63%)	16 (73%)	0.511
Undercorrection (exodeviation ≥10 PD)			
Postoperative 1 week	0 (0%)	3 (14%)	
Postoperative 1 month	1 (5%)	3 (14%)	
Postoperative 6 months	6 (32%)	6 (27%)	
At last follow-up	7 (37%)	6 (27%)	
Overcorrection (esodeviation ≥5 PD)			
Postoperative 1 week	0 (0%)	1 (5%)	
Postoperative 1 month	0 (0%)	1 (5%)	
Postoperative 6 months	0 (0%)	0 (0%)	
At last follow-up	0 (0%)	0 (0%)	

UMR: unilateral medial rectus; BMR: bilateral medial rectus; PD: prism dioptres; ^a^mean last follow-up time = 17.5 months ± 12 months (6–46 months).

**Table 3:** Examples for calculating amount of MR muscle resection.

Amount of exotropia (PD)	Unilateral or bilateral	Total amount of MR muscle resection (5 PD per 1 mm + 0.5 mm per muscle resected)	Total MR muscle resection per eye (mm)
55	Bilateral	11 + 1.0 mm	6
40	Bilateral	8 + 1.0 mm	4.5
20	Unilateral	4 + 0.5 mm	4.5
15	Unilateral	3 + 0.5 mm	3.5

PD: prism dioptres, MR: medial rectus.
